# Information theoretical quantification of cooperativity in signalling complexes

**DOI:** 10.1186/1752-0509-3-9

**Published:** 2009-01-16

**Authors:** Tom Lenaerts, Jesper Ferkinghoff-Borg, Joost Schymkowitz, Frederic Rousseau

**Affiliations:** 1 SWITCH, VIB, Brussels, Belgium; 2 Vrije Universiteit Brussel, Brussels, Belgium; 3 Ørsted.DTU, Technical Uiversity of Denmark, Kgs. Lyngby, Denmark

## Abstract

**Background:**

Intra-cellular information exchange, propelled by cascades of interacting signalling proteins, is essential for the proper functioning and survival of cells. Now that the interactome of several organisms is being mapped and several structural mechanisms of cooperativity at the molecular level in proteins have been elucidated, the formalization of this fundamental quantity, i.e. information, in these very diverse biological contexts becomes feasible.

**Results:**

We show here that Shannon's mutual information quantifies information in biological system and more specifically the cooperativity inherent to the assembly of macromolecular complexes. We show how protein complexes can be considered as particular instances of noisy communication channels. Further we show, using a portion of the p27 regulatory pathway, how classical equilibrium thermodynamic quantities such as binding affinities and chemical potentials can be used to quantify information exchange but also to determine engineering properties such as channel noise and channel capacity. As such, this information measure identifies and quantifies those protein concentrations that render the biochemical system most effective in switching between the active and inactive state of the intracellular process.

**Conclusion:**

The proposed framework provides a new and original approach to analyse the effects of cooperativity in the assembly of macromolecular complexes. It shows the conditions, provided by the protein concentrations, for which a particular system acts most effectively, i.e. exchanges the most information. As such this framework opens the possibility of grasping biological qualities such as system sensitivity, robustness or plasticity directly in terms of their effect on information exchange. Although these parameters might also be derived using classical thermodynamic parameters, a recasting of biological signalling in terms of information exchange offers an alternative framework for visualising network cooperativity that might in some cases be more intuitive.

## Background

A cellular pathway, whether enzymatic or signal transducing, can in a simplistic manner be described as a causal relationship between an environmental signal (such as nutrients, osmolytes or hormones) and a cellular response (generally through gene regulation). Cellular signals are mediated through a series of successive protein-protein interactions that bridge spatial and topological boundaries (between the plasma membrane and cell nucleus for example) and that allow for crosstalk between different pathways [1,2]. This protein-based modular strategy achieves integrated cellular responses that are both specific and at the same time tuned to global environmental and cellular requirements. This specificity is organized through the cooperativity between the members of the complex and the introduction of temporal and spatial constraints on the expression levels of the different members of the signalling pathways. Over- or under-expression, for instance, of the signalling components may have disastrous effects on the cellular phenotype, e.g. the development of cancer.

**Figure 1:**
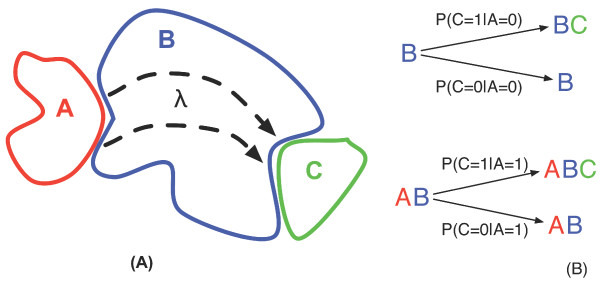
**Abstract ternary protein complex**. **(A) **The protein B in this abstract complex acts as a communication pathway between the two other proteins A and C. Binding protein A sends information over the pathway λ to the binding site of protein C, facilitating the binding of protein C. **(B) **Like a communication channel, the ternary complex can be described by a number of conditional probabilities. The conditional probabilities P(C = 0|A = 0) and P(C = 1|A = 1), describe the accuracy of the communication channel, i.e. the likelihood that a given output signal corresponds to the appropriate input signal. A second set of probabilities, P(C = 1|A = 0) and P(C = 0|A = 1), describes the intrinsic noise of the communication channel, i.e. the likelihood that a given input signal is not correctly conveyed.

Cooperativity is a thermodynamic concept that is used in different biochemical contexts [3-6]. Here this notion refers to the formation of multi-protein complexes with non-additive free-energies of assembly, i.e. complexes for which the stability of the final assembly is higher than the sum of all individual binary association [6]. A classic way to study cooperativity is by the analysis of a thermodynamic cycle [7]. Consider an assembly process that involves three proteins *A*, *B *and *C *that together form a ternary complex *ABC*, where *B *acts as an adaptor protein providing a separate binding surface for each of the two other molecules (see left panel Figure 1). Two alternative routes can then create the complex *ABC*: Either the binary complex *AB *is formed first and *C *binds afterwards or first the proteins *B *and *C *are joined before *A *is added. Every pair-wise reaction between the proteins in isolation and between individual proteins and partially formed complexes is annotated by a rate of dissociation (*Kd*), specifying the likelihood of that particular assembly/disassembly step. Here there are four dissociation constants: *Kd*_*A*-*B*_, *Kd*_*B*-*C*_, *Kd*_*AB*-*C *_and *Kd*_*A*-*BC*_, where the latter dissociation constants refer to the dissociation of *C *from *ABC *and *A *from *ABC *respectively. Since it is known that the overall thermodynamics and free energies for both routes to construct *ABC *is the same around the cycle (*Kd*_*A*-*B *_*Kd*_*AB*-*C *_= *Kd*_*B*-*C *_*Kd*_*A*-*BC*_), one can simply determine the cooperativity of the system by comparing the energy changes when one of the proteins is already bound or not [6]. For instance, when the likelihood of binding of *C *is enhanced when *A *is already bound to *B*, then there is positive cooperativity, resulting in bigger gain in energy when comparing it with the same reaction without the presence of *A*. When the binding of *C *to *B *is inhibited by the presence of *A*, then there is negative or anticooperativity, producing in turn a decrease in the free energy when comparing it to the process without *A*. When the presence of *A *in the complex does not influence the binding affinity of *C*, then there is no cooperativity or independent binding.

Even though this scheme identifies the presence and type of cooperation in the assembly process, it does not shed light on the molecular concentrations, possibly reflecting the intracellular conditions, required for efficient regulation or coordination between a pathway's active (*ABC*) and inactive (*B*) state. Here we provide an information theoretical method that, in the same spirit as the Hill and Scatchard plots [8], identifies and quantifies cooperativity in macromolecular assemblies and visualizes for a spectrum of concentrations when optimal coordination is obtained for the given experimental data. Different from those established methods, our approach goes beyond multiple bindings of the same ligand to a homogeneous oligomer (as in the binding of oxygen to haemoglobin [9]): We consider here the construction of heterogeneous protein assemblies mediated by multiple binding surfaces on adaptor proteins. As such, and as far as we are aware, this method provides an original and novel approach for the analysis of the cooperativity in macromolecular complexes that are part of some signalling cascade.

## Results and discussion

### General description of the approach

In analogy with cellular pathways, each protein in a cellular network can be considered as an element receiving an input signal (from upstream ligands) and generating an output signal (towards downstream effectors). Hence, we can reinterpret the ternary protein complex *ABC *as an instance of communication over a noisy channel [10,11], where protein *B *provides the communication channel through which information is exchanged between upstream ligand *A *and downstream effector *C *(See left panel in Figure 1). Like all noisy communication channels [10,11] the protein complex *ABC *is determined by a set of conditional probabilities relating input to output (See right panel in Figure 1). A first set of probabilities, *P(C = 0|A = 0) *and *P*(*C *= 1|*A *= 1), describe the accuracy of the communication channel, i.e. the likelihood that a given output signal corresponds to the appropriate input signal. In other words, if no input signal is given, meaning *A *is not bound to *B *(*A = 0*) then *C *should also be not bound to *B *(*C = 0*), and vice versa. A second set of probabilities, *P(C = 1|A = 0) *and *P*(*C *= 0|*A *= 1), describes the intrinsic noise of the communication channel, i.e. the likelihood that a given input signal is not correctly conveyed. Thus inappropriate transmission occurs when either *C *is bound to *B *(*C = 1*) even though *A *was not bound before (*A = 0*) or when *A *is bound (*A = 1*) and *C *is not (*C = 0*). These probabilities describe a partition of the initial protein concentrations *[A]*_0_, *[B]*_0 _and *[C]*_0 _over all possible association states of the system including *A*, *B *and *C *in isolation, the binary complexes *AB *and *BC *and the ternary complex *ABC*. Next to the total concentrations *[A]*_0_, *[B]*_0 _and *[C]*_0_, the steady state that is defined by this partitioning depends on the different dissociation constants including *Kd*_*A*-*B*_, *Kd*_*B*-*C*_, *Kd*_*AB*-*C *_and *Kd*_*A*-*BC*_. Given the steady state concentrations, the individual and conditional probabilities may be obtained (see Equation (1) in Methods). These probabilities are necessary to derive Shannon's entropy (*H(A)*, *H(C)*, ...) and subsequently the mutual information *I(A;C) *exchanged between *A *and *C *over channel *B *(see Equation (3) in Methods).

We quantify the degree of cooperativity of the system by the amount of information that is exchanged between the elements of the complex. In terms of the protein concentrations, this mutual information expresses how well the ratio of the steady-state concentrations of the ternary complex *ABC *and the free adaptor protein *B *are balanced while at the same time requiring the concentration of both binary complexes (*AB *and *BC*) to be as low as possible. So, on the one hand, low information exchange corresponds to an equilibrium situation where the protein (*B*) and complex concentrations (*ABC*) are out of balance or where to many intermediate complexes are present making it hard for the biological system to perform its function. On the other hand, high amounts of information exchange correspond to an optimized system where all members achieve the required coordination to switch efficiently between active and inactive states of the cellular process. Note, that the approach described here for a ternary protein complex can be further generalised to describe communication channels having multiple inputs or outputs (i.e. to study signal integrators or differentiators). In that case the mutual information between the different components needs to be deduced by a multivariate approach (see Methods) [11,12]. It is also important to note that the mutual information does not change from swapping the input with the output components, i.e. *I(A;C) = I(C;A)*.

### Biophysical model system

To clarify the biophysical meaning and illustrate our method we here describe the information exchange over a part of the p27 regulatory pathway. The p27 pathway controls the degradation of the cyclin-dependent kinase 2 (Cdk2) inhibitor p27 [13-16] thereby playing an important role in cell cycle progression [17,18]. In particular, phosphorylation of p27 triggers Cks1-mediated binding of p27 to Skp2. As Skp2 is part of the SCF^Skp2 ^ubiquitin ligase this results in p27 degradation and cell cycle progression. In a recent study [19], the assembling mechanism for part of the SCF^Skp2 ^multiprotein complex has been analyzed in order to understand 1) how and in which order the different units assemble and 2) how the specific order of this process influences the mutual affinities between the components and intermediately formed complexes. Seeliger et al. [19] showed that the Skp2-Cks1 complex increases the affinity of Cks1 for the Cdk2 inhibitor p27 a 100-fold. Additional inclusion of Cdk2 increases the affinity for p27 even more. Through mutational analysis the authors also showed long-range coupling between distant functional sites in Cks1, making it a principal example how adaptor proteins can play a central role in tightly controlling the assembly of a critical complex. As a consequence, it forms a biophysically meaningful case to investigate the communication between the different binding sites of the Cks1 structure in terms of Shannon's information theory (see Methods). Note here that Shannon's information theory can also be used to derive the communication pathway in Cks1. We recently demonstrated this lower-level analysis for the SH2 domain of Fyn [20]. Given the appropriate structural data, the same analysis could occur which should reveal the communication between the three binding sites [21].

The biophysical data obtained in [19], i.e. the dissociation constants, is used to perform the current analysis, (see also Table 1 for the data). The thermodynamic cycle including the adaptor protein Cks1 (acting as component *B*) [21], the proteins Skp2 (acting as component *C*) and p27 (acting as component *A*) produced from this data shows that both paths around the cycle are cooperative: Having Skp2 bound to Cks1 makes it easier for p27 to bind and vice versa. In a first step, we focus on the thermodynamic cycle for the formation of this ternary complex p27-Cks1-Skp2 (see Methods). Since *in vivo *p27 is bound to Cdk2, we will in a second step consider the quaternary complex Cdk2-p27-Cks1-Skp2. In that case two signals (Cdk2 and p27) are integrated and conveyed over the communication channel Cks1. As the mutual binding affinities of this system, i.e. *Kd*_*Skp*2-*Cks*1_, *Kd*_*Cks*1-*p*27_, *Kd*_*Skp*2-*Cks*1*p*27 _and *Kd*_*Skp*2*Cks*1-*p*27_, have been determined experimentally (see [19] and Table 1), we can quantify the information exchange between the input and output components of the system and study the transmission efficiency, meaning under which conditions we observe the highest degree of cooperativity, of the adaptor protein Cks1 under a wide range of chemical potentials (see Methods). Note that only one of the dissociation constants, *Kd*_*Skp*2-*Cks*1*p*27 _or *Kd*_*Skp*2*Cks*1-*p*27_, is required for the derivation of the different steady-state concentrations (see Methods).

**Table 1:** Dissociation constants between Cks1 and the different subunits.

**Protein or complex**	**Binding partner**	**Kd (μM)**
Cks1	p27	**70**

Cks1-Skp2	p27	**0.47**

Cks1-Cdk2	p27	**120**

Cks1-Cdk2-Skp2	p27	**0.15**

Cks1	Skp2	**0.014**

Cks1-Cdk2	Skp2	0.07

Cks1-p27	Skp2	0.0001

Cks1-Cdk2-p27	Skp2	0.0001

Cks1	Cdk2	**1.5**

Cks1-Skp2	Cdk2	8

Cks1-p27	Cdk2	3

Cks1-Skp2-p27	Cdk2	**2.6**

Dissociation constants in bold were derived experimentally. The remaining values were obtained through the thermodynamic cycles [19].

### How much information is exchanged in the p27-Cks1-Skp2 complex?

**Figure 2:**
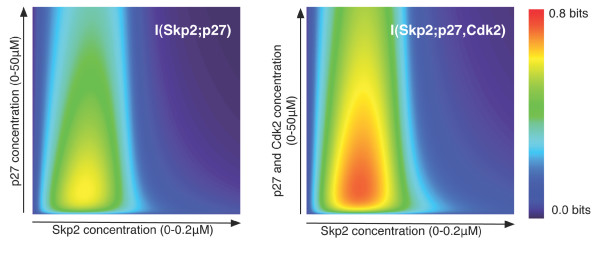
**Phase-space of cooperativity for the Cks1 adaptor protein**. Both contour diagrams show the mutual information for different concentrations of Cks1, Skp2 and phosphorylated p27 (left panel) and for different concentrations of Cks1, Skp2, Cdk2 and phosphorylated p27 (right panel). In both panels, the concentration of Cks1 is kept fixed (*0.1 μM*) and the concentration of Skp2 and p27 vary between *0.0–0.2 μM *and *0.0–50 μM *respectively. In the right panel, Cdk2 varies together with p27, meaning that we assumed *[p27] *= *[Cdk2] *for all combinations. As can be observed, the signal between Skp2 and phosphorylated p27 is clearly constrained by the input concentrations. Moreover, when adding Cdk2, as shown in the right plot, the signal is reinforced.

Figure 2 (left panel) shows the phase-space of the degree of cooperativity of the ternary p27-Cks1-Skp2 complex as it represents the Cks1-mediated information exchange between p27 and Skp2 over a range of concentrations varying between *0.0 μM *and *50 μM *for p27 and *0.0 μM *and *0.2 μM *for Skp2, whereas the concentration of Cks1 has been kept constant at *0.1 μM*. For each concentration distribution *{[p27], [Skp2]} *(Both ranges for Skp2 and phosphorylated p27 were discretized into 100 values each) the mutual information is calculated (see Methods), producing a matrix of information values. As argued earlier, mutual information expresses to what extent the proteins in the assembly properly coordinate their actions to achieve efficient switching between active and inactive states. Concretely when information about the association between Skp2 and Cks1 proteins is independent of the association of p27 and Cks1 proteins, then mutual information *I(Skp2, p27) *equals 0 bits (no cooperativity), turning the matrices in Figure 2 completely blue when this is the case. Conversely, when the association of all Skp2 proteins with Cks1 uniquely defines the associations of phosphorylated p27 then mutual information *I(Skp2, p27) *equals 1 bit (full cooperativity). This would mean that no binary assemblies are present, which is for most biochemical equilibria unlikely due to the underlying kinetics. Thus the intracellular process needs to find a good balance between the degree of cooperativity and the effectiveness of the switching mechanism.

As can be seen in Figure 2, only a small part of the phase space displays some or significant degree of cooperativity. In the cooperative regime the assembly of Cks1 to p27 will be conditional on the concentration of Skp2 and vice versa. In other words, binding of p27 to Cks1 and recruitment to the SCF^Skp2 ^ubiquitin ligase machinery will be mutually dependent events. Outside of this regime the assembly of these elements is still possible, but as under these conditions the fraction of bound protein is no longer influenced by changes in the concentration of the other, information exchange becomes very noisy. Moreover, from the difference in the extent for which each protein, Skp2 and p27, shows information exchange one can derive that it is Skp2 that forms the natural input signal for this regulatory process: It controls the switching in an effective manner.

### How does channel concentration affect robustness of the system?

**Figure 3:**
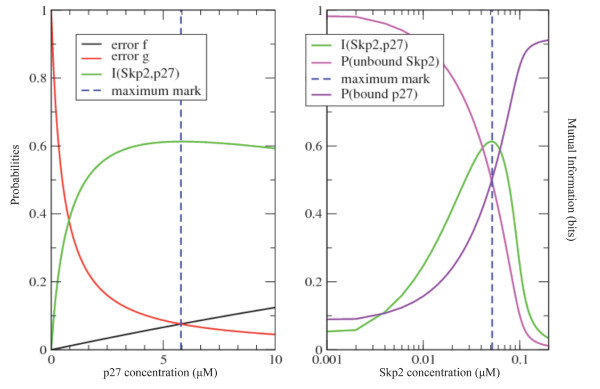
**Analysis of information exchange at Cks1 capacity**. In both plots, the mutual information (green line) is shown. In the left panel, it is visualized for optimal [Cks1]* and [Skp2]* and varying *[p27]*. In the right panel, the same information is shown for optimal [Cks1]* and [p27]* and varying *[Skp2]*. In both plots, the blue striped line marks the concentrations of p27 (left panel) and of Skp2 (right panel) where the channel's capacity is obtained. In the left panel, the error probabilities *f *and *g *are added and are shown to be equal when the optimal value of mutual information is achieved. Both error probabilities intersect around *f *= *g *= 0.075 for *[p27] *≈ *5.79 μM*. In the right panel, the probabilities that Skp2 is not bound to Cks1 and p27 is bound to Cks1 were added.

Interestingly, although the area of maximum cooperativity of p27-Cks1-Skp2 represents only a minor part of the phase space, it displays a relatively slow decline for increasing p27 concentration (see also Figure 3. left panel). This relatively broad, although suboptimal response curve, gives a measure for the robustness of the system to extrinsic noise due to fluctuations in input or output ligand concentrations. This is not the case for Skp2. As can be seen Figure 3 (right panel), the responsive area for Skp2 is very tightly defined (given *0.1 μM *concentration for Cks1). Hence variations in Skp2 strongly influence the responsiveness of the biochemical system.

**Figure 4:**
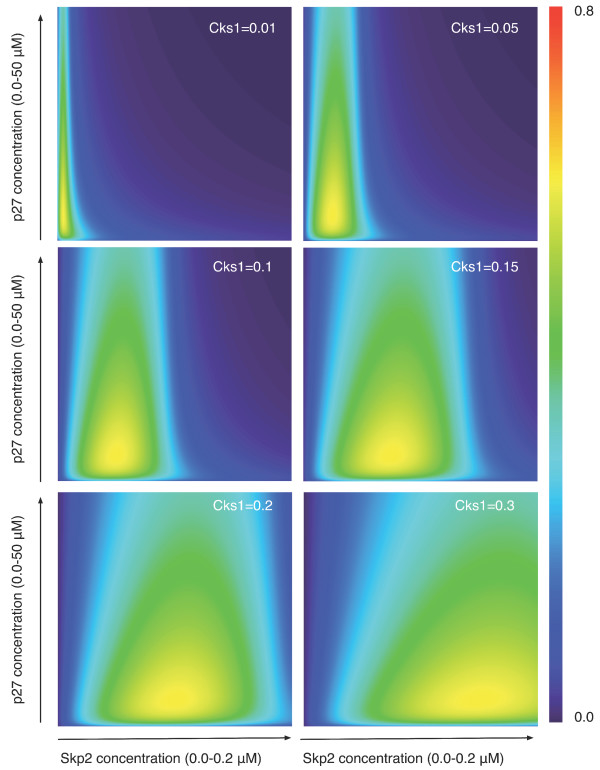
**Cooperativity phase-space for other concentrations of Cks1**. This figure extends the results shown in Figure 2, left panel. We show here that increasing the concentration of Cks1 (from *0.01 μM *to *0.3 μM*) results in an increase of cooperativity of the system, specifically for the concentrations of Skp2. Yet this increase has no effect on the maximum amount of information that is exchanged.

The robustness towards the Skp2 concentration increases as the concentration of Cks1 increases, as is shown in Figure 4. Increasing the concentration of Cks1 from *0.01 μM *and *0.3 μM *results in an increase of the cooperative area, particularly for the concentration of Skp2. This effect of Cks1 on the concentration of Skp2 makes sense biochemically since it is know that the expression of Cks1 alternates in parallel with the concentration of Skp2 when passing through the cell cycle [21]. Even though the area for Skp2 increases, the concentrations of p27 for which cooperativity is high remains the same. Moreover, even though the actual concentration for Skp2 changes, the maximum mutual information, or the *capacity*, remains always the same for this ternary system (see Figure 4).

### How much information is exchanged in the Cdk2-p27-Cks1-Skp2 complex?

**Figure 5:**
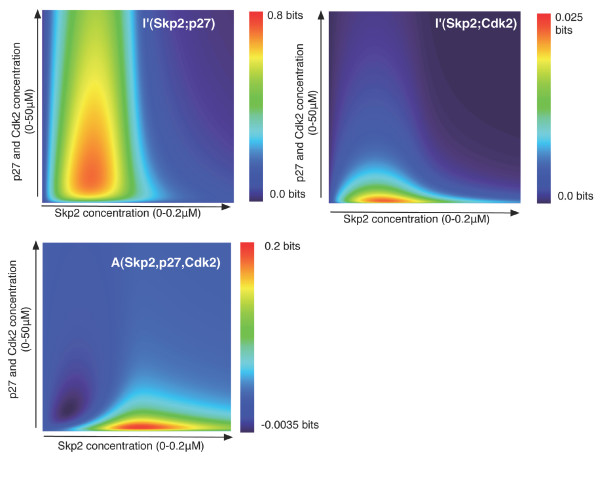
**Deconstruction of the information exchange in the quaternary complex Cdk2-p27-Cks1-Skp2**. This figure shows the mutual information between Skp2 and p27 (top left panel), Skp2 and Cdk2 (top right panel) and the effect of integrating both signals called mutual information (bottom left panel). The majority of the transmission occurs between the proteins Skp2 and p27. There is little interaction between Skp2 and Cdk2. Yet the stochiometric relation between p27 and Cdk2 modulates the signal in such a way that more information can be exchanged.

The right panel of Figure 2 shows the cooperativity profile of the quaternary complex Cdk2-p27-Cks1-Skp2. In this case both Cdk2 and p27, which are associated with a 1:1 stochiometry can be considered as two input signals, which are combined over the Cks1 adaptor to elicit Skp2 binding. The multivariate approach to mutual information [11,12] (see Methods) makes it possible to analyse the different components that define the information flow in this quaternary complex. As can be expected, the integration of the two signals gives rise to an increased cooperativity of the system, but also to a broader maximum indicating a more robust system response (see Figure 2, right panel). In addition, we can deconstruct the transmission within the complex Cdk2-p27-Cks1-Skp2 into the mutual information between Skp2 and p27 (Figure 5, top left panel), Skp2 and Cdk2 (Figure 5, top right panel) and the effect of integrating both signals, called the *interaction information A(Skp2, Cdk2, p27) *[12] (Figure 5, bottom left panel). Even though the majority of the communication occurs between Skp2 and p27, the integration of these two signals, *A *(see Methods), shows how the concentration of Cdk2 affects the communication between Skp2 and p27: First for low initial concentrations of both proteins, knowledge about Cdk2 inhibits slightly the information exchange over the cooperative channel. As the concentration of Skp2 increases, the transmission is amplified resulting in the higher capacity that is shown in the right panel of the Figure 2. Consequently, the contour plot of *A(Skp2, Cdk2, p27) *gives a quantitative interpretation of how these three proteins affect the communication over the channel Cks1.

### Channel capacity and noise of the p27-Cks1-Skp2 complex

The maxima of these contour diagrams represent the capacity of the system, i.e. the maximum amount of information that can be transmitted over the channel *with an arbitrary small probability of error *[22]. As can be seen in Figure 2 (left panel) channel capacity is achieved for the optimal input distribution {[p27], [Skp2]}* = {5.8 μM, *0.0512 μM} *(relative to the channel concentration [Cks1]* = *0.1 μM*) for the ternary complex p27-Cks1-Skp2. At these concentrations ~*0.61 *bits of information is received as output for every bit of input. As previously argued, this capacity remains the same even when the concentration of Cks1 changes. In terms of cooperativity, this means that even though the system is not fully cooperative, the balance between the assembled degradation system and independent adaptor protein is rather efficient while at the same time the intermediate complexes are few. Hence the capacity of the system is limited by the molecular concentrations that can be attained at steady state.

**Figure 6:**
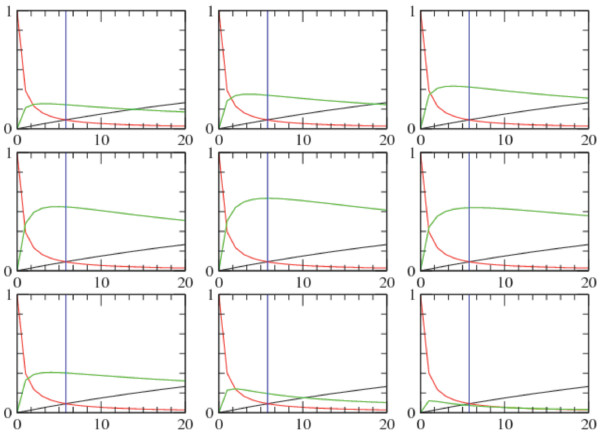
**Skp2 regulates the mutual information and noise in the communication**. We analysed the relationship between the mutual information for fixed concentrations Cks1. The concentration of Skp2 varies between plots from *0 μM *and *0.2 μM*. The concentration of p27 varies in each plot between *0 μM *and *20 μM*. Following the plots from top left to bottom right, the amount of Skp2 increases. As can be observed, the amount of mutual information (green line) also increases until some maximum is reached (centre plot). At this same point the error probabilities *f *and *g *intersect.

Figure 3 (left panel), shows further that optimal capacity is achieved when the error probabilities *f *and *g *are approximately equal, making the channel symmetric. The error probabilities intersect at *f *= *g *= 0.075 for *[p27] *= *5.8 μM*. This fact follows from our previous argumentation that the highest mutual information is attained when both the ratio of complete assembly and individual adaptor protein is well balanced and the concentrations of binary complexes are as low as possible. This small error value shows that the cooperative channel within Cks1 is a very efficient channel. Even more, for increasing values of *[Skp2]*, the capacity of the channel will never go beyond this point. This result is shown in Figure 6. When following the sequence of plots from the top left to the bottom right, one can observe that, while the concentration of Skp2 increases, magnitude of the mutual information (green line) increases until it reaches a maximum (centre plot in Figure 6). Afterwards the magnitude decreases again. Additionally we visualized the two error-probabilities *f *(black line) and *g *(red line) representing the error of reading a signal when no input was given (when p27 binds to Cks1 without prior binding of Skp2) and the error of giving a signal and not reading the output (when Skp2 binds to Cks1 without posterior binding of p27). As can be seen the concentration of Skp2 has no (or very little) influence on these probabilities since there is almost no change in both lines when comparing the different plots. The concentration of p27, on the other hand, has a strong influence on these values. When *[p27] *= *0 μM*, *f *becomes 0 and *g *becomes 1 (see Methods). For very high values of *[p27] *(like *50 μM*) the value of *f *reaches almost 50%, which indicates that the channel is completely random, and *g *becomes almost zero. This extreme situation is due to an over-representation of the p27 protein. As a consequence, there is more than enough p27 to bind to Cks1 without having any input signal (Skp2).

We added in each plot a blue line that marks the concentration of p27 where the maximum information transmission is found ([p27]* = *5.79 μM *and [Skp2]* = *0.0512 μM*) for all combinations of *[Skp2] *and *[p27] *(see also Figure 2 for this maximum). We immediately notice here that this vertical line lies at the intersection of the two error probabilities (left panel Figure 3) as well as the *50% *mark of the probability of having p27 bound to Cks1 (right panel Figure 3). Consequently, when the channel is symmetric the SCF^Skp2^model system studied here reaches maximal information exchange.

By adding Cdk2 in the system, the capacity becomes ~*0.75 *bits (see Figure 2 right panel), confirming the effect of including Cdk2 as discussed in ref. 10. Moreover, the area of optimal response increases (see red area in Figure 2 right panel) allowing a wider range of useful Skp2 and p27 concentrations.

## Conclusion

All these results show that, given the binding affinities at equilibrium and the overall concentrations of the different components, mutual information quantifies for which protein concentrations the systems' cooperativity, or more specific its coordination, is optimal. Our analysis clearly shows (see Figure 2) that both Skp2 and Cks1 concentrations are crucial parameters to obtain proper regulatory behaviour in the p27 degradation system. If binding is independent then no information exchange would be observed. Moreover, this narrow range for Skp2 remains operational for a wide range of p27 concentrations, making the SCF^Skp2 ^ubiquitination system robust to variations in p27 abundance. When moving outside the boundaries defined for Skp2 the coordination between the complex members is lost, leading possibly to continuous activation without proper regulation. This observation seems to corroborate experimental studies on the over expression of Skp2 (and Cks1) in relation to the development cancer [23-26]. Even though, the current analysis is performed on the steady-state information of this biochemical system, the underlying idea is that all the proteins of the macromolecular complex become expressed so that their mutual concentrations fall into the highly cooperative area (see Figure 2). Further analysis is off course required to verify this hypothesis. To conclude, the present result show that Shannon's information theory quantifies the cooperativity of biochemical systems, making it an important tool for the current attempts to understand cooperativity in a systems perspective.

## Methods

### Defining Cks1 as an asymmetric noisy channel

In information theory communication occurs through noisy channels[10], where the noise is the result of an error in the transmission. Different kinds of channels exist, but here the focus is on *discrete and memoryless channels*. Concretely, a noisy channel is defined by an input alphabet *A*_*X*_, output alphabet *A*_*Y*_, a set of conditional probability distributions *P(y|x)*. The conditional probability distributions provide for every input signal (*x *∈ *A*_*X*_) the probability that a particular output signal (*y *∈ *A*_*Y*_) is produced. When the alphabets contain only two symbols and the probability of having a miscommunication is the same for both input symbols, the channel is also referred to as a *binary symmetric noisy channel*. Given this description, the cooperative pathway within Cks1 can now be defined as a noisy channel where the input and output alphabets both consist of the symbols *0 *and *1*, referring respectively to the unbound and bound states of both binding sites of Cks1. To keep things simple, the channel description of Cks1 uses only two of the three proteins that bind to Cks1 in the SCF^Skp2 ^model system: Skp2 and phosphorylated p27. Since the symbols refer to bound and unbound state of either Skp2 or p27 to Cks1, there are four probabilities relevant here: The probability that both Skp2 and p27 are bound, that both are unbound and that Skp2 is bound (unbound) to Cks1 and p27 is unbound (bound). These probabilities are visualized in Figure 1 (right panel). Since the information transmission corresponds to one of the four output complexes in the right panel of Figure 1, the associated probabilities can be derived from the concentrations of these complexes at equilibrium. These probabilities can be organized in a transmission matrix *Q*:

(1) $$\left(\begin{array}{cc} 1-f & g\\ f & 1-g \end{array}\right)$$

where *f *and *g *are defined as

$$\begin{array}{lll} f & = & P\left(p27=1|Skp2=0\right)\\ & = & \frac{\left[Cks1-p27\right]}{\left[Cks1]+[Cks1-p27\right]}\\ g & = & P\left(p27=0|Skp2=1\right)\\ & = & \frac{\left[Skp2-Cks1\right]}{\left[Skp2-Cks1]+[Skp2-Cks1-p27\right]} \end{array}$$

This relation between concentrations implies that the errors, and later also the information exchange, depends on the concentration of the proteins that may be produced by the system.

### Determining the equilibrium concentrations

We determine the concentrations of the different proteins and protein complexes using the dissociation constants determined by Seeliger et al. [19]. Using these binding affinities a system of equations is derived, which is numerically solved by determining the roots of these equations. For the simplified model system, which only incorporates Cks1, Skp2 and phosphorylated p27, this system of six equations is the following:

(2) $$\begin{array}{ll} 1. & {\left[Cks1\right]}_{0}=[Cks1]+[Cks1-p27]+\\ & \left[Skp2-Cks1]+[Skp2-Cks1-p27\right]\\ 2. & {\left[Skp2\right]}_{0}=[Skp2]+[Skp2-Cks1]+\\ & \left[Skp2-Cks1-p27\right]\\ 3. & {\left[p27\right]}_{0}=[p27]+[Cks1-p27]+\\ & \left[Skp2-Cks1-p27\right]\\ 4. & Kd_{\left[Skp2-Cks1\right]}=\frac{\left[Cks1]*[Skp2\right]}{\left[Skp2-Cks1\right]}\\ 5. & Kd_{\left[Cks1-p27\right]}=\frac{\left[Cks1]*[p27\right]}{\left[Cks1-p27\right]}\\ 6. & Kd_{\left[Skp2Cks1-p27\right]}=\frac{\left[Skp2-Cks1]*[p27\right]}{\left[Skp2-Cks1-p27\right]} \end{array}$$

The system contains six parameters, namely *[Cks1]*_0_, *[Skp2]*_0_, *[p27]*_0_, *Kd*_ [*Skp*2-*Cks*1]_, *Kd*_ [*Cks*1-*p*27] _and *Kd*_ [*Skp*2*Cks*1-*p*27]_. The first three parameters correspond to the total concentrations of the proteins in the model system both in isolation and in complexes. The latter three parameters are the three dissociation constants specific to the SCF^Skp2 ^model system. Note that the dissociation constant *Kd*_ [*Skp*2*Cks*1-*p*27] _refers here to the dissociation of *p27 *from the complex *Skp2-Cks1-p27*. The results remain the same if the alternative dissociation constant, dissociating *Skp2 *from *Skp2-Cks1-p27*, is used. When the values for these parameters are inserted from ref. 19, a root finding algorithm is applied to determine the equilibrium concentrations of all the members of this system: *[Cks1]*, *[Skp2]*, *[p27]*, *[Skp2-Cks1]*, *[Cks1-p27] *and *[Skp2-Cks1-p27]*. Once these concentrations are obtained, the probabilities in the matrix *Q *can be determined.

### Calculating mutual information

Mutual information expresses the amount of information that the output conveys about the input (and vice versa). It is formally expressed in terms of entropy:

(3) $$\begin{array}{c} I(X;Y)=I(Y;X)\\ =H(X)+H(Y)-H(X,Y)\\ =H(X)-H(X|Y) \end{array}$$

where the entropies are calculated as:

$$\begin{array}{l} H(X)=-\sum_{x\in X}p(x)\log p(x)\\ H(X,Y)=-\sum_{x,y\in X,Y}p(x,y)\log p(x,y)\\ H(X|Y)=-\sum_{y\in Y}p(y)\left(\sum_{x\in X}p(x|y)\log p(x|y)\right) \end{array}$$

The base of the logarithm determines the units in which mutual information is expressed. Usually it is either a natural (*ln x*) or a binary logarithm (*log*_2 _*x*), making the units either nats (natural digits) or bits (binary digits). Here, a binary logarithm is used. So mutual information (see Equation 3) expresses how much we learn about the output (or input) of a channel when we receive information about the input (or output). This is calculated by subtracting the entropy (uncertainty) on the state of the output (or input) from the entropy (uncertainty) of the output (or input) when we know the input (or output). So all entropy scores are related to the state of the channel (here Cks1) and not the state of the input and output proteins, respectively Skp2 and p27.

Concretely, all entropy values can be easily derived from the probabilities related to the input and output state of Cks1. For instance, if *X *corresponds to Skp2, then *P(Skp2 = 0) *and *P(Skp2 = 1) *correspond to the probabilities that Skp2 is bound or not bound to Cks1. This leads to the following formulation of the entropy for Skp2:

(4) $$\begin{array}{l} H(Skp2)=\\ -\sum_{i\in \{0,1\}}P(Skp2=i)\log\left(P(Skp2=i)\right) \end{array}$$

where

$$\begin{array}{l} P\left(Skp2=0\right)=\frac{\left[Cks1]+[Cks1-p27\right]}{\Theta }\\ P\left(Skp2=1\right)=\frac{\left[Skp2-Cks1]+[Skp2-Cks1-p27\right]}{\Theta }\\ \Theta =[Cks1]+[Skp2-Cks1]+[Cks1-p27]\\ +[Skp2-Cks1-p27] \end{array}$$

The entropy *H(p27) *is derived in the same way. The joint entropy is

(5) $$\begin{array}{l} H(Skp2,p27)=\\ -\sum_{i,j\in \{0,1\}}P(Skp2=i,p27=j)\log\left(P(Skp2=i,p27=j)\right) \end{array}$$

where

$$\begin{array}{l} P\left(Skp2=0,p27=0\right)=\frac{\left[Cks1\right]}{\Theta }\\ P\left(Skp2=1,p27=0\right)=\frac{\left[Skp2-Cks1\right]}{\Theta }\\ P\left(Skp2=0,p27=1\right)=\frac{\left[Cks1-p27\right]}{\Theta }\\ P\left(Skp2=1,p27=1\right)=\frac{\left[Skp2-Cks1-p27\right]}{\Theta } \end{array}$$

### Multivariate mutual information

To derive the information exchange between three or more proteins a multivariate approach needs to be followed[11,12]. This approach allows the analysis of the signal between two input proteins and an output protein. As in the previous formulation, the mutual information is determined using entropy:

(6) $$\begin{array}{c} I(X;Y,Z)=I(Y,Z;X)\\ =H(X)+H(Y,Z)-H(X,Y,Z) \end{array}$$

where X represents the output signal and Y and Z represent two input signals or visa versa. In addition, the effect of either one of the components on the two other ones can be analysed by eliminating this component. For instance if one wants to determine the effect of Cdk2 on the communication between Skp2 and phosphorylated p27, the mutual information *I(Skp2;p27) *and the averaged transmitted information *I*_*Cdk*2_*(Skp2;p27) *need to be determined (see ref. 9 for the details). If *I(Skp2;p27) *is not equal to *I*_*Cdk*2_*(Skp2;p27) *then Cdk2 has an effect on the transmission between the two other proteins. This difference, called the interaction information *A(Skp2, Cdk2, p27)*, is the gain (or loss) in the sample information transmitted between any two of the proteins, caused by the additional knowledge of the third one. Combining the interaction information with *I(Skp2;p27) *and *I(Skp2;Cdk2) *produces again the multivariate mutual information *I(Skp2; Cdk2, p27)*. As a consequence, *A(Skp2, Cdk2, p27) *expresses how the two signals are modulated, which can be either in a negative of positive way.

## Authors' contributions

TL carried out the mathematical modelling and contributed in the conception and development of the principles of this work. JFB, JS and FR conceived the principles of the work and assisted in the development of the mathematical model. TL, JS and FR drafted the manuscript. All authors read and approved the manuscript.
